# Crohn’s Disease and Ulcerative Colitis: From Pathophysiology to Novel Therapeutic Approaches (3rd Edition) †

**DOI:** 10.3390/biomedicines14051068

**Published:** 2026-05-08

**Authors:** Dingpei Long, Junsik J. Sung

**Affiliations:** 1 State Key Laboratory of Resource Insects, Institute of Sericulture and Systems Biology, Southwest University, Chongqing 400715, China; 2 Digestive Disease Research Group, Center for Inflammation, Immunity & Infection, Institute for Biomedical Sciences, Georgia State University, Atlanta, GA 30303, USA; 3 Department of Pharmaceutical Sciences, School of Pharmacy, University of Maryland, Baltimore, MD 21250, USA; 4 Department of Pharmaceutical and Administrative Sciences, College of Pharmacy and Health Sciences, Western New England University, Springfield, MA 01119, USA

## 1. Introduction

The first editorial in this series approached inflammatory bowel disease (IBD) predominantly from the perspective of pathophysiology and therapeutic innovation [1]. The second shifted the emphasis toward precision and patient-centered care [2]. The papers collected in this third edition show why this transition has become necessary. Although Crohn’s disease (CD) and ulcerative colitis (UC) remain the two defining clinical categories of IBD, current research continues to show that these labels explain only part of what clinicians encounter in practice. Disease course, treatment response, complication patterns, and long-term outcomes are more variable than the traditional CD/UC framework alone can adequately explain.

This broader heterogeneity runs through this Special Issue. Read together, these papers do more than update familiar topics. They show where the field is moving. The central question is no longer only which pathways drive inflammation, or which new drug has entered the therapeutic landscape; increasingly, the more relevant question is how to connect mechanisms, risks, and therapies more accurately in individual patients. In this sense, this third edition brings pathophysiology, clinical complexity, and treatment strategy into the same frame.

## 2. Looking Beyond Broad Diagnostic Labels

One of the clearest messages from this Special Issue is that IBD cannot be reduced to a single inflammatory pathway. The reviews on gut microbiota and mucosal immune homeostasis, microbiome-based therapies in UC, fibrostenotic Crohn’s disease, and the therapeutic effects of distinct molecular inhibitors all point in the same direction: epithelial injury, dysbiosis, immune activation, mesenchymal remodeling, and defective repair are closely linked [3,4,5,6]. This view is consistent with the broader direction of the field. Recent guidelines and major reviews increasingly treat IBD less as a uniform inflammatory disorder and more as a group of related conditions requiring mechanism-aware and context-sensitive treatment decisions [7,8,9,10,11,12].

The renewed interest in microbiota-directed therapy belongs to the same shift. The review on microbiome-based therapies in UC is especially timely because it moves beyond descriptive discussions of dysbiosis and focuses on intervention [4]. By comparing fecal microbiota transplantation with defined microbial consortia, and by placing both within a precision-oriented framework, it shows how microbial ecology is beginning to move from background mechanism to therapeutic target [13,14,15]. This is an important change. It suggests that future treatment strategies may depend not only on blocking downstream inflammatory signals, but also on restoring ecological and metabolic functions that have been lost.

This Special Issue also widens the biological frame in other ways. A single-cell transcriptomic analysis identified an opioid-signaling-high monocyte subset linked to TNF-related communication in inflamed IBD tissue [16]. This finding is notable not only for its mechanistic implications, but also because it reminds us that pathways often discussed in relation to pain or symptom control may also play a direct part in organizing inflammation. A study of psychological profiles in UC and CD makes a similar point from a different angle [17]. Disease is not experienced only through endoscopy, histology, or biomarkers. Emotional and behavioral patterns can shape how symptoms are felt, interpreted, and managed. Additional work in this Special Issue on infection risk associated with biologics and proton pump inhibitors, COVID-19-related receptor biology and vaccine serological responses, and the immunological overlap between IBD and type 2 diabetes extends the discussion further toward host vulnerability and systemic comorbidity [18,19,20,21]. Taken together, these papers argue for a broader and more realistic understanding of IBD biology, one that includes psychosocial, infectious, metabolic, and host–environment dimensions alongside mucosal inflammation.

## 3. Where the Hardest Clinical Questions Still Remain

Another strength of this Special Issue is its attention to the parts of IBD care that remain most difficult. Post-operative recurrence in Crohn’s disease continues to remind clinicians that surgery, however necessary, is not curative [22]. Chronic inflammatory pouch disorders remain similarly challenging. The multinational study included here showed low long-term persistence of first-line anti-TNF therapy and a substantial rate of pouch failure in chronic inflammatory pouch conditions [23]. This matters because it reinforces a point that is sometimes easy to overlook: pouch-related inflammation cannot always be managed as though it were simply luminal UC or CD in another form.

Acute severe ulcerative colitis (ASUC) presents a different kind of difficulty, defined less by chronic complexity than by speed and consequence. In this setting, there is often little room for prolonged therapeutic trial and error. The review in this Special Issue captures this reality well, particularly the move from conventional rescue paradigms toward a broader advanced-therapy framework [24]. Recent work outside this Special Issue points in the same direction. Vedolizumab has further defined its place in chronic pouchitis [25,26], while newer rescue data with tofacitinib and the reassessment of intensified infliximab induction have sharpened the discussion around treatment selection in steroid-refractory ASUC [27,28,29].

These are not isolated examples. They reflect a broader change in IBD research and practice. Some of the most clinically useful progress is now coming not from studies of broad IBD populations, but from work focused on the highest-risk subgroups: patients with post-operative recurrence, pouch disorders, severe colitis, fibrosis, fistulizing disease, or repeated biologic failure. In such settings, the key question is often not which treatment is generally effective, but which treatment best fits a particular biological and clinical context.

## 4. From More Options to Better Treatment Matching

This Special Issue also reflects how much the therapeutic landscape has expanded. Reviews on JAK inhibitors, sphingosine-1-phosphate receptor modulators, microbiome-based therapies, and broader molecular inhibitor strategies make clear that IBD treatment is no longer defined by a simple step-up sequence of progressively stronger drugs [4,6,30,31]. In practice, therapeutic selection now depends on much more: disease phenotype, prior biologic exposure, anticipated speed of response, route of administration, safety profile, extraintestinal burden, and patient preference [9,29,32,33,34,35,36,37,38].

Even so, the most important change may not be the number of available options, but the beginning of better treatment matching. Several papers in this Special Issue point clearly in this direction. A machine learning model for anti-drug antibody formation during infliximab induction in Crohn’s disease, an observational study of cytokine dynamics during ustekinumab induction, and a pharmacogenomic analysis of vedolizumab response all share a common logic [39,40,41]. Their value lies not in claiming that one marker can predict everything, but in showing that treatment response is increasingly being approached as something that may be anticipated, at least in part, rather than judged only after failure has become obvious.

This same movement can be seen across the wider literature. Recent advances in single-cell and spatial profiling, immunogenicity genetics, blood-based markers, pharmacoepigenetics, dual-target strategies, and multi-omics frameworks are steadily reshaping what precision medicine in IBD may realistically mean [42,43,44,45,46,47,48,49]. At the same time, the discussion has become more practical. The field is now paying closer attention to clinical trial design, advanced combination treatment frameworks, and the infrastructure needed to turn biological knowledge into usable treatment decisions [50,51,52]. Even the inclusion here of a review on gene therapy in Crohn’s disease fits this broader trajectory [53]. Gene-based treatment remains preclinical and faces major translational barriers. Even so, its presence in this Special Issue is meaningful because it reflects a field that is beginning to think beyond repeated cycles of inflammatory suppression and toward the possibility of more durable local reprogramming.

This, ultimately, is what gives this third edition its coherence. Across studies of microbiota, fibrosis, infection risk, psychological profiles, pouch disorders, ASUC, drug immunogenicity, cytokine pharmacodynamics, pharmacogenomics, and gene therapy, the message is not that IBD has become simple. It is that the field is getting better at recognizing that patients who appear similar diagnostically may differ substantially in biology, risk, and therapeutic need. The next challenge is to turn this sharper biological reading into more reliable treatment selection. This Special Issue does not close this gap, but it does show clearly where the gap now lies.
